# Case report: Changes in defense mechanisms, personality functioning, and body mass index during psychotherapy with patients with anorexia nervosa

**DOI:** 10.3389/fpsyg.2023.1081467

**Published:** 2023-02-20

**Authors:** Ciro Conversano, Mariagrazia Di Giuseppe, Vittorio Lingiardi

**Affiliations:** ^1^Department of Surgical, Medical and Molecular Pathology, Critical and Care Medicine, University of Pisa, Pisa, Italy; ^2^Department of History, Culture and Society, University of Rome Tor Vergata, Rome, Italy; ^3^Department of Dynamic and Clinical Psychology, and Health Studies, “Sapienza”, University of Rome, Rome, Italy

**Keywords:** eating disorders, defense mechanisms, personality, body mass index, psychotherapy

## Abstract

Eating disorders (EDs) are difficult to treat in psychotherapy due to their pervasive symptomatology and frequent and rapid relapses. Restrictive anorexia nervosa (AN) is the most challenging ED, often associated with severe physical and mental conditions. Perceived as an ego-syntonic syndrome that somehow protects the patient from a number of developmental tasks, treating AN requires extensive multidisciplinary long-term intervention. As with other emotion regulation strategies, defense mechanisms mediate an individual's reaction to internal or external stressors, including those related to ED conditions. Improving defensive functioning adaptiveness predicts psychotherapy outcomes and is an essential component of the therapeutic process. In this study, we qualitatively described changes in the use of defense mechanisms, personality functioning (PF), and body mass index (BMI) in two patients with severe AN in treatment with intense dynamic psychotherapy. Changes in personality functioning and defense mechanisms were periodically assessed every 6 months using clinician report measures such as the Shedler-Westen Assessment Procedure-200 (SWAP-200) and the Defense Mechanisms Rating Scales Q-sort (DMRS-Q), respectively. BMI was also monitored throughout the treatment. A qualitative description of the patient's defensive profile and the quantitative score on all ranges of defense mechanisms were used for studying changes in patients' use of defenses during the treatment and relationships between defenses and outcome indexes. Personality and defensive functioning improved after 1 year of intense dynamic psychotherapy, independently from BMI improvement. All outcome indexes dramatically decreased before a scheduled interruption of the treatment, underling that an integrated therapeutic approach is essential for improving and eventually orienting toward complete ED symptoms remission. Long-term dynamic psychotherapy fosters self-awareness of psychological distress and enhances more mature ways of coping. Monitoring changes in personality and defense mechanisms helps in understanding patients' reactions to stressful life events and in developing specific therapeutic interventions.

## Introduction

Eating disorders (EDs) are one of the most insidious psychopathologies to treat due to the chronical symptomatology and frequent relapses during and after the course of psychotherapy (Grenon et al., 2019). Notably, when treating patients with EDs, clinicians report distinct patterns of negative emotional responses—-including strong feelings of ineffectiveness and incompetence, frustration, anger, and lack of mutual connection—-which negatively affect the therapeutic process and outcome (e.g., Thompson-Brenner et al., 2012; Tanzilli et al., 2020). Among EDs, anorexia nervosa (AN) is the most challenging and worrying clinical condition with significantly high mortality (Papadopoulos et al., 2009; Franko et al., 2013; Gugliandolo et al., 2020). Despite indisputable research advances, the rate of patients with AN completely recovered is still very low. Among surviving patients, less than one-half recover on average, whereas one-third improve and 20% remain chronically ill (Steinhausen, 2002). It is worth noting that often patients with AN report being afraid of changes required by the treatment (psychological, medical, nutritional, and psychiatric integrative therapy) and tend to feel their pathology as protective against developmental tasks and growth (Garner and Garfinkel, 1980). Given the partial effectiveness of treatments for EDs, advances in etiology understanding and treatment tailoring are needed to improve therapeutic outcomes with patients with AN (Hay et al., 2012; Zipfel et al., 2015).

Defense mechanisms are documented to be one of the most relevant among psychological factors influencing therapeutic outcome (Di Giuseppe et al., 2020a; Giovanardi et al., 2021). Defense mechanisms are an important aspect of implicit emotion regulation that moderates psychological reactions to internal conflicts and external stressful situations, such as chronic physical and mental diseases (Conversano et al., 2020; Vicario et al., 2020; Vita et al., 2020; Martino et al., 2021), defined as mechanisms that mediate the individual's reaction to internal or external stressors (American Psychiatric Association, 2013). Research suggested that the use of immature defenses is associated with psychopathology, whereas mature defenses are related to high psychological functioning (Lucifora et al., 2021; Di Giuseppe et al., 2022). Moreover, a large body of literature demonstrated that stressful life events predict worsening in defensive functioning, together with symptom exacerbation and a higher level of psychological distress (Aafjes-Van Doorn et al., 2021; Békés et al., 2022). This is particularly disturbing for patients affected by chronic diseases, whose defense mechanisms mediate the course of the disease and survival probability (Beresford et al., 2006).

Research on defensive functioning of patients with AN still scarce, and controversial findings have been found. Gothelf et al. have found that patients with AN rely more on immature defenses compared with controls (Gothelf et al., 1995). Defenses such as intellectualization and sublimation are more frequently used by patients with AN than healthy people, while passive aggression, isolation, and devaluation are more frequently used by anorectic than bulimic patients (Stein et al., 2003; Costanzo et al., 2022). These results were not confirmed by other studies. According to Vidović et al., patients with AN display no significant difference in defense styles when compared with healthy patients, which the authors explained as the tendency to control external and internal environment and the unconscious efforts to imitate normality to avoid conflicts (Vidović et al., 2003).

Patients' use of defense mechanisms has been shown to be an important predictor of treatment outcomes. Over the course of the treatment, patients' defenses improve as they tend to use more mature defenses and less neurotic and immature defenses (Kramer et al., 2010; Babl et al., 2019). The changes in defensive functioning do not follow necessarily a linear trend, they are generally influenced by emotionally meaningful life events and the quality of significant relationships, such as the one with the therapist or important others (Tanzilli and Gualco, 2020). Improvement in defensive maturity is also associated with improvement in personality functioning and self-awareness. In this regard, it is plausible to define mature defenses as coping strategies (i.e., the defense affiliation is similar to problem-focused coping), therefore reducing the ancient dichotomy between these psychological constructs (Di Giuseppe et al., 2021a).

According to recent findings, high-adaptive defense mechanisms sustain functional personality development in healthy individuals (Costa et al., 1991; Cramer, 2015; Muzi et al., 2021). The in-deep understanding of defense mechanisms on a continuum of maturity/adaptiveness (Vaillant, 1971) requires the reference to the gold-standard interpretation of this psychological aspect of mental functioning: the Defense Mechanisms Rating Scales (DMRS), an empirically based hierarchical theory of defense mechanisms that inspired the introduction of an axis for assessing defenses in the DSM-IV (Perry, 1990). The hierarchical organization of defense mechanisms proposed in the DMRS includes 30 defense mechanisms ordered into seven defense levels and three defensive categories of maturity. Different from other theories of defense mechanisms, the DMRS hierarchy of defenses highlights both the definition and the function of each defense mechanism and describes clinical examples of how each defense operates (Di Giuseppe and Perry, 2021). Analyzing the function of defense mechanisms is possible to understand the internal conflicts experienced by the patient and, thus, address therapeutic intervention.

In this study, we examined changes in defensive functioning during long-term psychotherapy with two patients diagnosed with severe AN. The changes in defensive functioning have been qualitatively described together with the body mass index (BMI) and personality functioning (PF). We aimed to verify whether changes in overall defensive functioning (ODF) and specific defense mechanisms together with BMI and PF can be detected during intense dynamic psychotherapy with patients having AN. We expected that high-adaptive defense mechanisms such as self-assertion and self-observation would be associated with improvement in BMI and PF, while image-distorting defenses such as splitting and devaluation of self-image would be associated with a decrease in both outcome indexes.

## Methods

### Participants and procedure

In the present study, we describe two case reports of patients with restrictive AN treated with integrative therapy for ED that included nutritional therapy, medical monitoring, drug therapy, and psychotherapy. The psychotherapeutic setting consisted of three sessions per week provided by a private practitioner dynamic psychotherapist with more than 10 years of experience. For both patients, nutritional therapy was already provided at intake, while drug therapy started few months after the beginning of psychotherapy treatment. A professional net of clinicians was promptly built and coordinated by the psychotherapist to support the patient's adjustment to expected changes and control possible symptoms relapses. The frequency of nutritional, medical, and psychiatric appointments was deliberately decided by doctors according to symptom severity, while the frequency of psychotherapy sessions remained stable for the entire course of the treatment.

The first case, referred to using the pseudonym Daphne, was a 16-year-old girl with severe restrictive AN that emerged around 13 years of age. At the time of entering psychotherapy, she was attending the 11th grade of a very demanding and time-consuming high school in a small city in Central Italy. She reported having tried a couple of previous psychotherapies that she stopped attending after a few months. The psychotherapy reported in the present study lasted 3 years, including a 6-month interruption for ED residential treatment. The second case, referred to by the pseudonym Maria, was a 45-year-old divorced woman with a severely restrictive AN that had emerged at around 40 years of age. She graduated in economics with top grades and worked for 10 years as a business manager of a local factory. Meanwhile, she engaged for many years in a romantic relationship with a colleague whom she left as the AN became pervasive in her life. After a few months, she also quit her job and focused exclusively on her illness. She tried a number of psychological interventions, but all ended after few sessions because of her fear of emotional engagement. The psychotherapy described in the present study lasted 18 months. The patient dropped out after a 1-month planned interruption for due to medical problems of her therapist. The drop-out was indirectly announced by a gradual decline in the patient's mental functioning a month before the therapist's hospitalization. Both patients were the first of two sisters, born in upper-class families, and educated with high cultural and ethical standards. Perfectionisms, self-devaluation, fear of being criticized, and disengagement in social relationships were psychological aspects common to both patients. A history of distancing and cold relationships with patients, psychological abuse, and humiliation was also reported by both patients.

Psychological functioning and BMI were systematically monitored every 6 months. Patients were asked to certify their agreement in being part of a research study by signing an informed consent. All procedures were conducted in accordance with the ethical standards outlined in the Declaration of Helsinki.

### Measures

The Defense Mechanisms Rating Scale Q-sort (DMRS-Q; Di Giuseppe et al., 2014) is an observer-rated measure for the assessment of defense mechanisms in the clinical setting. Based on the gold-standard theory of defense mechanisms (Perry, 1990), the DMRS-Q asks the clinician to rank-order 150 statements into a seven-rank forced distribution, which is helped by the computerized DMRS-Q software available online at https://webapp.dmrs-q.com. As with other DMRS-based measures, the DMRS-Q provides scores for 30 defense mechanisms, seven defense levels, and an index of overall defensive maturity. In addition, the DMRS-Q provides the Defensive Profile Narratives (DPN), a qualitative description of the patient's defensive profile based on the most representative defensive patterns used by the individual (Tanzilli et al., 2021). The DMRS-Q showed moderate to excellent reliability for both trained (ICC_ODF_ = 0.90) and untrained coders (ICC_ODF_ = 0.88). Good criterion validity was found when comparing the DMRS-Q to the original DMRS (Békés et al., 2021).

The Shedler Westen Assessment Procedure-200 (SWAP-200; Shedler and Westen, 2004) is a personality assessment method designed to provide clinicians with a standard “vocabulary” for case description. It consists of 200 statements, each of which may describe a given patient very well, somewhat, or not at all. The clinician describes a patient by rank-ordering 200 items into eight categories of descriptiveness (higher scores mean higher patient descriptiveness). The SWAP-200 provides scores for 12 factors: psychological health, psychopathy, hostility, narcissism, emotional dysregulation, dysphoria, schizoid orientation, obsessionality, thought disorder, oedipal conflict (histrionic sexualization), dissociation, and sexual conflict. These factors showed strong convergent and discriminant validity, and the median inter-rater reliability of SWAP diagnostic scales is above 0.80 (Shedler and Westen, 2004). In the present study, we used the Italian version of the SWAP-200 (Shedler et al., 2014) and included psychological health factors as an outcome measure.

The Body Mass Index (BMI; World Health Organization, 1995) is a measure of body fat that is the ratio of the weight of the body in kilograms to the square of its height in meters. Four categories were established to measure an individual's weight status: underweight (BMI ranging from 15 to 19.9), normal (BMI ranging from 20 to 24.9), overweight (BMI ranging from 25 to 29.9), and obese (BMI ranging from 30 to 35 or greater). For children and young people aged 2–18, the BMI calculation considers age and gender as well as height and weight.

### Statistical analyses

Descriptive statistics and changes over time in ODF, PF, and BMI were reported using means, standard deviations, and delta variations.

## Results

### Changes over time in ODF, PF, and BMI

**Figure 1:**
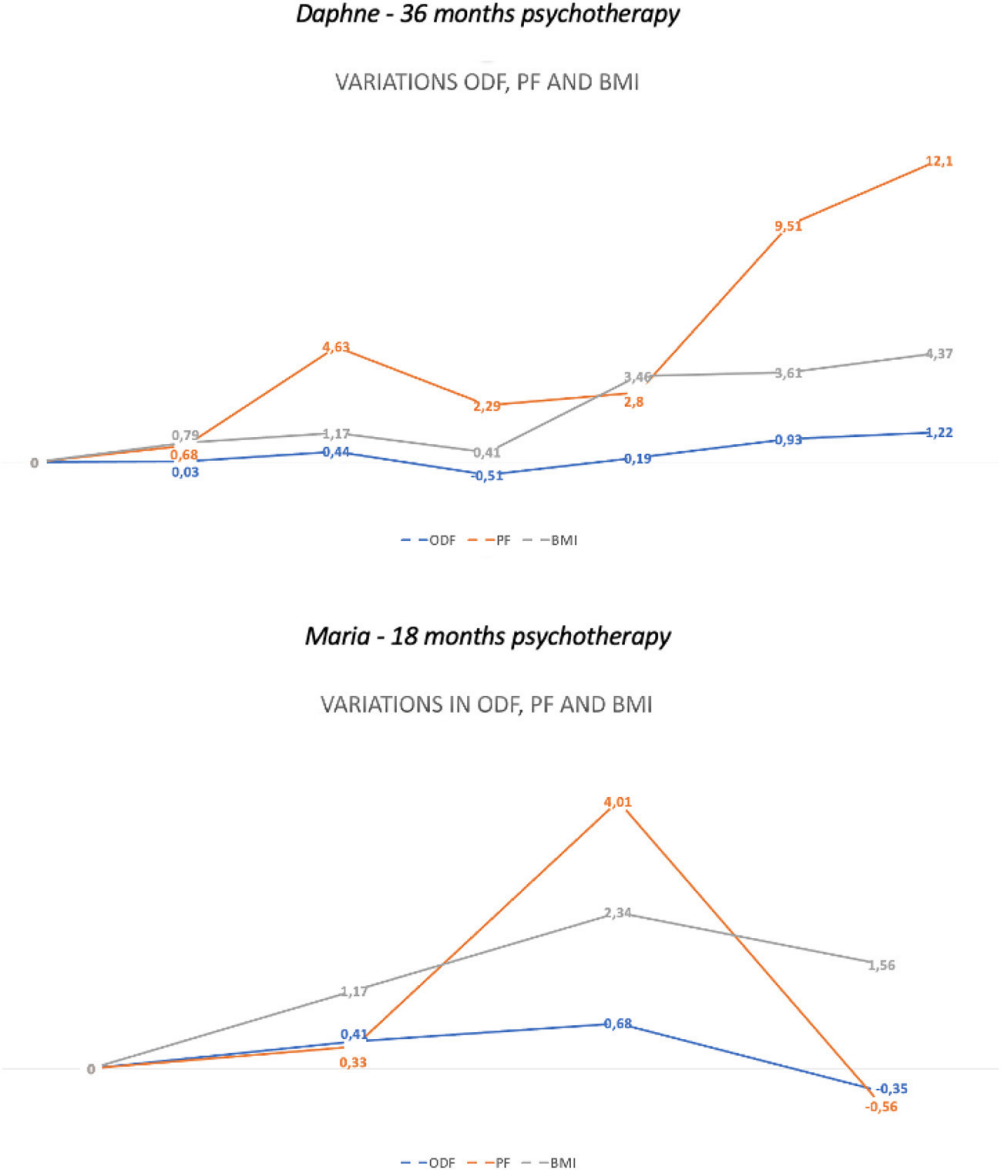
Changes in ODF, PF, and BMI during psychotherapy.

The changes in ODF, PF, and BMI over time are displayed in Table 1. Both cases have shown an improvement in all outcome indexes after 1 year of intense dynamic psychotherapy, which dramatically decreased before a scheduled interruption of the treatment (Daphne's Δ_*ODF*_ = −0.51; Maria's Δ_*ODF*_ = −0.35). In one case (Daphne), the patient went back to psychotherapy after a 6-month interruption for residential treatment for ED and reached complete recovery from AN symptoms after 1 more year of intense psychotherapy. In the other case (Maria), the patient dropped out after the interruption in a situation of acute AN symptoms relapse (Figure 1). Improvement in outcome measures followed different trends. While BMI was mostly affected by nutritional therapy (increase of 3.4 in BMI after 6 months of residential treatment), ODF and PF were highly affected by psychotherapy (increase of 3.4 in BMI after 6 months of residential treatment). However, the interruption of one of these treatments determined a stalled condition for overall recovery, while the interruption of both treatments determined severe symptoms relapse (see values at T4 in Figure 1).

**Table 1:** Descriptive statistics of changes in ODF, PF, and BMI over the course of psychotherapy.

		**Raw scores**			Δ **from intake**		
		**ODF**	**PF**	**BMI**	**ODF**	**PF**	**BMI**
**Daphne (recovered case)**							
ODF	Intake: start psychotherapy	4.59	49.34	14.8	-	-	-
	6th month: psychotherapy	4.62	50.02	15.6	0.03	0.68	0.8
	12th month: psychotherapy	5.03	53.97	16.0	0.44	4.63	1.2
	18th month, before separation	4.08	51.63	15.2	−0.51	2.29	0.4
	24th after residential treatment	4.78	52.14	18.2	0.19	2.80	3.4
	30th month psychotherapy	5.52	58.85	18.4	0.93	9.51	3.6
	End: 36th month psychotherapy	5.81	61.44	19.2	1.22	12.10	4.4
**Maria (dropped out case)**							
ODF	Intake: start psychotherapy	4.56	50.23	16.0	-	-	-
	6th month: psychotherapy	4.97	52.56	17.2	0.41	2.33	1.2
	12th month: psychotherapy	5.24	56.24	18.4	0.68	6.01	2.4
	Drop out:18th month, before separation	4.21	49.67	17.6	−0.35	−0.56	1.6

## Discussion

Known as a key factor of personality structure, defensive functioning influences cognitive, affective, relational, and behavioral strategies activated by an individual in response to internal conflicts and external stressors (Lingiardi et al., 2010; Hersoug et al., 2021). Like other emotion regulation strategies, defense mechanisms tend to change throughout psychotherapy, and their improvement impact symptoms severity and overall mental health (Perry and Bond, 2012; Di Giuseppe et al., 2020b; de Roten et al., 2021; Perry et al., 2022). The present study described the variation of defensive functioning, body weight, and overall personality functioning in two cases of severe AN-treated integrated therapy for ED, including long-term dynamic psychotherapy. Qualitative and quantitative assessments of defense mechanisms have been done with a measure inspired by the gold-standard and empirically based theory of defense mechanisms (Perry, 1990), the Q-sort version of the DMRS (DMRS-Q) developed for assessing defenses in clinical work (Di Giuseppe et al., 2014, 2021b). Despite the evident limitations of the case study design, the present research represents a pioneer attempt at a systematic longitudinal assessment of defense mechanisms and BMI variations in severe AN. Moreover, it provides an example of how observer-rated measures such as the DMRS-Q might be easily applied in process-outcome research with limited costs (Békés et al., 2021).

Improvement in ODF, PF, and BMI was observed in both cases after 1 year of dynamic psychotherapy. Conversely, we observed decreased defensive maturity and symptom relapses right before scheduled therapy interruptions. These findings confirmed our hypothesis that improvement in defensive maturity was associated with an increase in BMI and PF. Interestingly, we found that only the patient who returned to psychotherapy after 6 months of interruption (Daphne) showed complete remission. In this case, the increase in outcome measures followed different trends depending on the treatment provided and the specific therapeutic setting. In the cases analyzed, the outpatient nutritional therapy setting was only slightly helpful for improving BMI, although it allowed the patient to continue her intense dynamic psychotherapy, which therefore fostered defensive and personality improvements. Residential treatment for ED dramatically impacted BMI but did not allow psychotherapy continuation, which resulted in a sort of “freezing” of psychological functions and outcomes. Regarding the defense mechanisms on the basis of our observation, it can be speculated that the ability to recur to high-adaptive defenses such as self-observation and self-assertion allowed patients with AN to understand, reflect, and actively take care of their illness with the effect of reducing maladaptive eating behaviors, obsessive thoughts, social isolation, and body misperception. Accordingly, using less repression and denial facilitated patients' awareness of their severe chronic disease instead of keeping them blind and disengaged from solving the problem. Moreover, less use of devaluation, splitting, and passive aggression decreased self-harming behaviors and pervasive self-image distortion with the effect of symptoms decrease and BMI increase. Looking at the specific function of defense mechanisms in these severe AN cases, there might be an inhibited awareness of conflictual ideas, thoughts, and desires (repression), possibly related to body perception and identity (devaluation of self-image), which led to an extreme negative image distortion that could not be mitigated by considering positive aspects of the self and the others (splitting). Dramatic chronic food restriction might be seen as a strategy for turning anger toward the self (passive aggression). However, this symptom is denied and instead justified as a fear of fat body shape (denial).

Moreover, we noted that residential treatment impacted BMI, while it “freezed” improvements in defensive and personality functioning. Different therapeutic interventions (i.e., residential therapy for ED, intense dynamic psychotherapy, and drug therapy) might work for specific purposes at specific times (Roth and Fonagy, 2005), but they should not be exchanged one for the other. They instead should be integrated when treating complex psychopathological conditions such as severe AN. The absence of an integrated approach or the poor intensity of the intervention might compromise the effectiveness of the whole therapeutic process and thus foster relapses, symptoms exacerbations, and chronicity.

### Study limitations

The present study has some important limitations that need to be considered. First, it analyzed only two cases of patients with severe AN in treatment, which cannot exhaustively represent this clinical population; second, demographic characteristics, quality of social support, and stressful life events were not been analyzed as potential risk factors affecting outcome; third, observer-rated measures were used after therapy sessions without the support of records and transcriptions, which impede the comparison with self-reported assessment of changes; and finally, changes in psychological and physical variables were monitored every 6 months without considering variation occurring within the entire psychotherapy process. Future studies should address these limitations by providing data from a larger sample of patients with AN treated with long-term psychotherapy and assessed with both self-report and clinician-report instruments. Research should consider sociodemographic characteristics and other psychosocial conditions as potentially impactful factors on changes during the treatment. Further process outcome studies should investigate session-by-session variations in symptoms and psychological resources in relation to overall psychical conditions.

## Conclusion

Treating ED is a long-term journey studded with pitfalls. Understanding psychological mechanisms behind symptom maintenance and relapses is essential to contrast dysfunctional patterns activated during the treatment with patients affected by severe AN. Effective therapeutic interventions should adopt the biopsychosocial approach to care and consider the daily monitoring of such mechanisms to lead the patient toward the genuine acceptance of changes in weight and body shape promoted by nutritional therapy.

## Data availability statement

The raw data supporting the conclusions of this article will be made available by the authors, without undue reservation.

## Ethics statement

Ethical review and approval was not required for the study on human participants in accordance with the local legislation and institutional requirements. Written informed consent to participate in this study was provided by the participants' legal guardian/next of kin. Written informed consent was obtained from the individual(s) and/or minor(s)' legal guardian/next of kin for the publication of any potentially identifiable images or data included in this article.

## Author contributions

CC conceived the research design and analyzed the data. MD wrote the first draft of the manuscript and collected data by coding the therapy sessions. CC, MD, and VL contributed to the interpretation of the results and critically reviewed the final draft of the manuscript. All authors contributed to the article and approved the submitted version.
